# Vitamin D3 Enhances Endothelial Function and Improves Vascular Reactivity in an Experimental Model of Type 2 Diabetes Mellitus in Rats

**DOI:** 10.3390/ph19060867

**Published:** 2026-05-30

**Authors:** Amanda Suellenn da Silva Santos Oliveira, Joyce Lopes Macedo, Lais Lima de Castro Abreu, Ana Karolinne da Silva Brito, Ana Victória da Silva Mendes, José Otávio Carvalho Sena de Almeida, Andressa Amorim dos Santos, José Vinícius de Sousa França, Janyerson Dannys Pereira da Silva, Daniel Dias Rufino Arcanjo, Maria do Carmo de Carvalho e Martins

**Affiliations:** 1 Department of Nutrition, Federal University of Piauí, Teresina 64049-550, PI, Brazil; amandasuellenn@hotmail.com (A.S.d.S.S.O.); lais.castro123@ufpi.edu.br (L.L.d.C.A.); 2 Department of Biophysics and Physiology, Federal University of Piauí, Teresina 64049-550, PI, Brazil; joycelopes385@gmail.com (J.L.M.); karolinnebrito@ufpi.edu.br (A.K.d.S.B.); victoriams18@hotmail.com (A.V.d.S.M.); otavios.almeida@hotmail.com (J.O.C.S.d.A.); amorimandressa092@gmail.com (A.A.d.S.); vinicius.sfranca@ufpi.edu.br (J.V.d.S.F.); janyerson.silva@ufpi.edu.br (J.D.P.d.S.)

**Keywords:** aorta, Diabetes Mellitus Type 2, endothelium vascular, rats Wistar, vascular reactivity, Vitamin D3

## Abstract

**Background/Objectives**: Type 2 Diabetes Mellitus (T2DM) is characterized by insulin resistance and chronic hyperglycemia, which significantly impair vascular function. In experimental T2DM models, the vascular endothelium is compromised, showing decreased vasodilator responses. Vitamin D3 has emerged as a promising intervention for improving glycemic parameters and restoring endothelial function. This study evaluated the effects of vitamin D3 (0.25 and 0.50 µg/kg/day) administered for 4 and 8 weeks on the ex vivo aortic vascular reactivity of T2DM rats. **Methods**: T2DM was induced in male Wistar rats via a high-fat, normoprotein diet and streptozotocin (30 mg/kg, i.p.). Groups included normal control, diabetic control, metformin, and vitamin D3 (0.25 or 0.50 µg/kg/day). Following 4 or 8 weeks of treatment, thoracic aortic segments were isolated for ex vivo vascular reactivity studies to assess responses to vasoconstrictor and vasorelaxant agents. **Results**: Vitamin D3 treatment improved glycemic profiles; the 0.25 µg/kg dose reduced fasting glucose, while the 0.50 µg/kg dose lowered glycated hemoglobin at 8 weeks. Endothelium-dependent relaxation induced by acetylcholine was significantly increased in diabetic rats treated with vitamin D3 at both doses over 4 weeks compared to diabetic controls. Moreover, vitamin D3 prevented the attenuation of maximal contractile responses to phenylephrine observed in untreated diabetic rats at 8 weeks. **Conclusions**: Vitamin D3 supplementation restores endothelial function and improves vascular reactivity in an experimental T2DM model. These findings suggest that vitamin D3 may mitigate vascular complications by enhancing vasorelaxation and maintaining contractile integrity.

## 1. Introduction

Type 2 Diabetes Mellitus (T2DM) is a chronic metabolic disorder that represents a major global health challenge, accounting for approximately 90% of all diabetes cases worldwide. It is characterized by persistent hyperglycemia and insulin resistance, which are primary drivers of systemic complications, particularly vascular dysfunction, and also involves changes in the secretion of incretin hormones [1].

In the long term, the metabolic alterations in DM2 lead to chronic complications, including microvascular complications such as retinopathy, kidney disease and neuronal damage, and macrovascular complications, particularly ischemic heart disease, cerebrovascular disease and peripheral vascular disease [2]. Furthermore, experimental models of T2DM consistently demonstrate significant compromise of the vascular endothelium, leading to impaired endothelium-dependent vasodilator responses, with reduced bioavailability of the endothelial mediators nitric oxide and prostacyclins, which are responsible for regulating blood flow [3].

Endothelial dysfunction is characterized by a decrease in the vasodilator response caused by the synthesis of relaxing factors, with a proven reduction in the magnitude and potency of muscarinic agonists in experiments in which vascular reactivity and any damage to endothelial physiology, organization and stability of the endothelium are investigated [2,4,5]. In DM2, hyperglycemia, insulin resistance and inflammation damage endothelial cells, inducing an imbalance between the production of vasodilator and vasoconstrictor factors [6,7].

Considering the metabolic changes and vascular complications in T2DM, there has been growing interest in evaluating the effects of nutritional interventions in T2DM [8,9]. In this context, Vitamin D3 has been extensively investigated for its potential role in modulating vascular health and improving endothelial function in metabolic disorders [10,11].

Vitamin D3 corresponds to a group of molecules called calciferols and is a fat-soluble vitamin, identified as a prohormone [12]. This vitamin has a dominant role in bone metabolism [13], but it also plays a role in the removal of xenobiotics, reducing oxidative stress, neural protection, antimicrobials, modulating the immune system, regulating inflammation, as well as anti-cancer properties, effects on insulin sensitivity and secretion and cardiovascular benefits [14].

With regard to endothelial function, studies in the literature describe that vitamin D3 promotes its actions through vitamin D3 receptors (VDR), which are expressed in vascular endothelial cells [15]. Vitamin D3 deficiency in animal models affects endothelium-dependent relaxation with decreased expression of the enzyme endothelial nitric oxide synthase [5]. In addition, the vasodilator response to acetylcholine is reduced in the absence of VDR in the endothelium [16]. In humans with DM2, low vitamin D3 concentrations have been associated with the presence of endothelial dysfunction [17,18].

Evidence also shows a significant improvement in vascular function in groups treated with vitamin D3 [19] and in the response to arterial vasodilators that depend on nitric oxide [20]. In this context, chronic treatment with vitamin D3 restores the characteristics of relaxation to acetylcholine in isolated arterial preparations and promotes a decrease in vascular tone, as well as reducing blood pressure values ([11]).

Cholecalciferol (vitamin D3) was selected as the supplemental form in preference to calcitriol [1,25(OH)2D3], the biologically active metabolite, owing to its wider therapeutic window and substantially lower risk of inducing hypercalcemia, making it more appropriate for long-term in vivo supplementation protocols. We hypothesize that daily supplementation with Vitamin D3 improves endothelium-dependent vasodilation and restores vascular reactivity by counteracting the damaging effects of chronic hyperglycemia. Therefore, this study aimed to evaluate the effects of daily vitamin D3 supplementation (0.25 and 0.50 µg/kg) for 4 and 8 weeks on the aortic vascular reactivity of rats with type 2 diabetes.

## 2. Results

### 2.1. Basic and Laboratory Parameters of the In Vivo Experimental Protocol

Initial body weights were similar in all study groups, and final weights presented a significant weight loss at 4 and 8 weeks. As shown in Table 1, Vitamin D3 at a dose of 0.50 µg/kg for 4 weeks promoted a lesser increase in the body weight gain when compared with CN1 and CD1 groups. On the other hand, 8 weeks of treatment with Vitamin D3 did not significantly attenuate this altered weight gain or restoration of the weights to control levels (Table 2). This phenotype is a hallmark of the HFD-STZ experimental model; the pathophysiological relevance of this reduced weight gain lies in the catabolic state induced by insulin deficiency and resistance, which leads to muscle wasting and adipose tissue mobilization, exacerbated by caloric loss through glycosuria. The persistence of this weight profile even under Vitamin D3 supplementation indicates that, while the treatment may modulate vascular parameters, it does not fully counteract the systemic metabolic exhaustion characteristic of severe T2DM in this model.

Blood glucose levels and the percentage of glycated hemoglobin at the end of the study were significantly higher in the diabetic groups compared to the normal control group, at both treatment times (Table 1 and Table 2). The groups treated with vitamin D3 at the lowest dose and at both treatment times had significantly lower mean fasting glycemia values than the CD groups (*p* < 0.05). On the other hand, the animals treated with vitamin D3 at a dose of 0.50 µg/kg/day had lower mean fasting glycemia values than the CD group in the 4-week treatment and lower mean glycated hemoglobin values compared to the CD animals in the 8-week treatment (*p* < 0.05). Interestingly, in the 8-week VIT D 0.50 group, Vitamin D3 treatment significantly reduced HbA1c levels (5.17 ± 1.28% vs. 8.22 ± 1.83% in CD, *p* < 0.05), despite fasting glucose remaining elevated (401.70 ± 16.47 mg/dL).

### 2.2. Functional Study of the Aorta Ex Vivo

**Figure 1:**
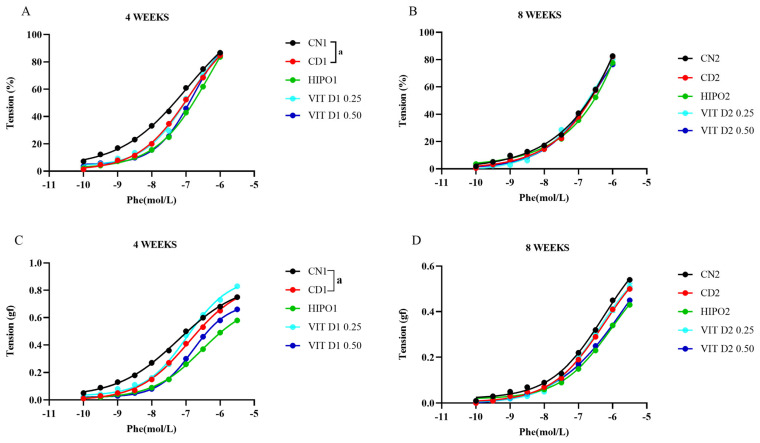
Concentration-effect relationship for the cumulative addition of phenylephrine, analyzed by the variation of tension as percentage (%) (**A**,**B**) or gram-force (gf) (**C**,**D**), in isolated thoracic aortic rings of rats treated with vitamin D3 at doses of 0.25 and 0.50 µg/kg/day for 4 weeks and 8 weeks, respectively). One Way ANOVA followed by Tukey’s test. Significant differences are presented as: ^a^ *p* < 0.05 when compared to CN. CN—non-diabetic rats; CD—diabetic rats not submitted to any treatment; HIPO—diabetic rats treated with metformin; VIT D 0.25—diabetic rats treated with vitamin D3 at a dose of 0.25 µg/kg; VIT D 0.50—diabetic rats treated with vitamin D3 at a dose of 0.50 µg/kg.

In this study, considering the analysis of the concentration and contractile response curves for phenylephrine by the variation in tension as a percentage of the maximum effect (E_max_), no significant differences were observed in the E_max_ and effective concentration at 50% of E_max_ (EC_50_) in the groups treated with vitamin D3 (0.25 and 0.50 µg/kg) for 4 and 8 weeks, compared to the CD group, indicating no changes in vascular contractility (Table 3 and Table 4). In the CD group, a reduction in the contraction response was observed only in isolated rings of the animals that received treatment for 4 weeks (Figure 1A,C).

When vascular contractility was assessed in grams (g), the contractions produced by phenylephrine were lower in aortic rings isolated from animals in the CD group under treatment for 4 weeks. The contractile responses obtained with the addition of phenylephrine in rings isolated from the group treated with vitamin D3 (0.25 µg/kg) did not differ significantly from the responses observed in rings from the CD group, despite the observation of a greater magnitude in the effect of phenylephrine at concentrations above 100 nmol/L (Figure 1C). The same analysis in arteries isolated from the group treated with vitamin D3 (0.25 and 0.50) for 8 weeks did not show any differences (Figure 1B,D).

**Figure 2:**
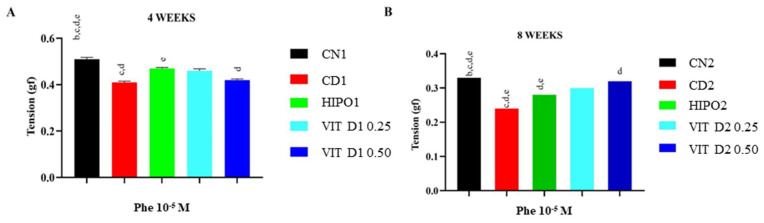
Magnitude of contraction in grams at a submaximal concentration of phenylephrine in isolated thoracic aortic rings of rats treated with vitamin D3 (0.25 and 0.50 µg/kg/day) for 4 (**A**) and 8 weeks (**B**). One Way ANOVA followed by Tukey’s test. Significant differences are presented as: ^b^ *p* < 0.05 when compared to CD; ^c^ *p* < 0.05 when compared to HIPO; ^d^ *p* < 0.05 when compared to VIT D 0.25; ^e^ *p* < 0.05 when compared to VIT D 0.50. CN—non-diabetic rats; CD—diabetic rats not submitted to any treatment; HIPO—diabetic rats treated with metformin; VIT D 0.25—diabetic rats treated with vitamin D3 at a dose of 0.25 µg/kg; VIT D 0.50—diabetic rats treated with vitamin D3 at a dose of 0.50 µg/kg.

Based on this observation, we evaluated the maximum contraction in grams produced by the addition of phenylephrine at a submaximal concentration (1 µmol/L) in the isolated rings of the groups studied. In this analysis, the response to phenylephrine corresponded to 0.51 g and 0.33 g in isolated rings from rats in the CN, 4- and 8-week treatment groups, respectively. There was a reduction in the magnitude of the contraction in the CD groups compared to the CN group (*p* < 0.0001), which corresponded to 0.41 g and 0.24 g for the 4- and 8-week groups. In the isolated arterial rings of the groups treated with vitamin D3 at a dose of 0.25 µg for 4 and 8 weeks, there was a magnitude of contraction corresponding to 0.46 g and 0.30 g and in the groups treated with a dose of 0.50 µg at both treatment times (4 and 8 weeks), the magnitude of contraction was 0.42 and 0.32, respectively. This response indicates a greater magnitude of contraction in these groups when compared to the CD groups (*p* < 0.0001), except in the group treated with vitamin D3 at the lowest dose for 4 weeks (*p* > 0.05) (Figure 2A,B). The magnitude obtained in the arterial rings of the animals treated with vitamin D3 for 8 weeks showed a relationship with the dose of vitamin D3 with which they were treated (*p* = 0.0117).

Together with this analysis, the E_max_ values calculated from the concentration-effect curves for phenylephrine in grams, carried out on rings isolated from the groups treated for 4 weeks, showed a reduction in E_max_ in the CD group (0.95 ± 0.07 g) compared to the CN (1.07 ± 0.21 g). In the group treated with vitamin D3 (0.25), similar response magnitudes were observed (0.98 ± 0.10 g) (Table 3).

In the experiments carried out to assess endothelial function, the cumulative addition of acetylcholine produced functional endothelium-dependent relaxations in rat thoracic aortic rings in all the groups studied. In the CN group, the E_max_ and EC_50_ values corresponded in grams: CN1 = 0.59 ± 0.04/4.18 × 10^−6^ ± 8.98 × 10^−7^; CN2 = 110.60 ± 8.85/5.50 × 10^−6^ ± 2.10 × 10^−6^; in percentage: CN1 = 117.14 ± 9.28/4.18 × 10^−6^ ± 8.98 × 10^−7^; CN2 = 5.50 × 10^−6^ ± 2.10 × 10^−6^, in rings isolated from rats at 4 and 8 weeks of treatment, respectively (Table 5 and Table 6).

The relaxation observed in the CD groups corresponded to E_max_ values of 0.30 ± 0.03 g and 82.25 ± 13.84% for CD1 and 0.25 ± 0.01 g and 73.52 ± 4.80% for CD2. With regard to EC_50_ values, mean percentage values were 4.27 × 10^−6^ ± 2.22 × 10^−6^ for CD1 and lower values for CD2 (2.29 × 10^−6^ ± 6.22 × 10^−7^), indicating a reduction in the endothelial response to stimulation with acetylcholine for both parameters analyzed (E_max_ and EC_50_).

**Figure 3:**
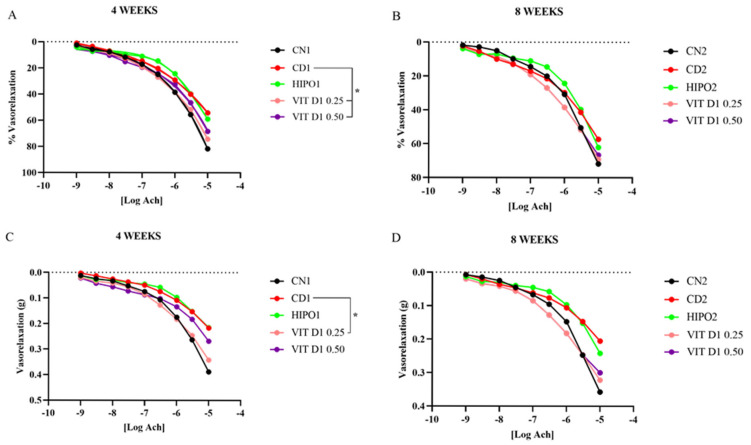
Concentration-effect relationship for the cumulative addition of acetylcholine in isolated thoracic aortic rings of rats precontracted with phenylephrine, analyzed by the variation of tension (%) (**A**,**B**) and relaxation in grams (**C**,**D**), and treated with vitamin D3 (0.25 and 0.50 µg/kg/day) for 4 and 8 weeks, respectively. One Way ANOVA followed by Tukey’s test. Significant differences are presented as: * *p* < 0.05 when compared to CD. CN—non-diabetic rats; CD—diabetic rats not submitted to any treatment; HIPO—diabetic rats treated with metformin; VIT D 0.25—diabetic rats treated with vitamin D3 at a dose of 0.25 µg/kg; VIT D 0.50—diabetic rats treated with vitamin D3 at a dose of 0.50 µg/kg.

The concentration-effect curves for acetylcholine obtained in isolated rings from rats treated with vitamin D3 for 4 weeks, at doses of 0.25 and 0.50 µg/kg, showed a higher E_max_ value (calculated from the data in percentage or grams of relaxation). Thus, vitamin D3 treatment improved the vasorelaxant profile at 4 weeks, with the 0.25 and 0.50 µg/kg doses significantly increasing the maximal relaxation (E_max_). The differences observed were significant compared to the values obtained in the CD group, in grams: VIT D1 0.25 = 0.49 ± 0.05; CD1 = 0.30 ± 0.03; *p* < 0.05; in percentage: VIT D1 0.50 = 137.80 ± 13.87; CD1 = 82.25 ± 13.84/*p* < 0.05) (Table 5). These data indicate that acetylcholine produced relaxations of greater magnitude, but without a significant change in potency, as can be seen from the EC_50_ values (*p* > 0.05). Furthermore, the response obtained by vasorelaxation in the group that received the lowest dose of vitamin D3 was close to the control curve (CN) (Figure 3A,C), which suggests that this dose may have caused a smaller reduction in endothelium-dependent relaxation.

In relation to the concentration-effect curves carried out on isolated arterial rings from rats belonging to the groups treated with vitamin D3 for 8 weeks, the results suggest the absence of an effect on endothelium-dependent relaxation induced by Ach when compared to the CD group at both doses, since the quantitative data obtained were similar between the groups (Figure 3B,D). However, these differed significantly from the data from the CN group for the highest supplementation dose, if the relaxations were analyzed as a percentage (%) (VIT 0.50 = 80.63 ± 6.85; CN = 110.60 ± 8.85) or in grams (VIT 0.50 = 0.39 ± 0.03; CN = 0.56 ± 0.04/*p* < 0.05), which may indicate that hyperglycemia reduced vasorelaxation and that vitamin D3 at this dose was not effective in blocking this effect (Table 6).

## 3. Discussion

The principal findings of this study demonstrate that vitamin D3 supplementation exerts dose- and time-dependent effects on glycemic control and aortic vascular function in the HFD-STZ rat model of T2DM. At 4 weeks, both doses significantly enhanced endothelium-dependent relaxation, an effect not sustained at 8 weeks, suggesting a temporal limitation of the vascular benefits under prolonged hyperglycemic conditions. A notable dissociation between fasting glycemia and HbA1c was observed at 8 weeks with the 0.50 µg/kg dose, consistent with the capacity of vitamin D3 to reduce non-enzymatic hemoglobin glycation independently of fasting glucose, likely through its antioxidant properties [21,22]. This time-dependent divergence in endothelial outcomes may reflect stabilization of circulating 25-OH vitamin D3 levels and consequent modulation of vitamin D3 receptor (VDR) expression and downstream signaling [23,24,25].

The reduced contractile response to phenylephrine in diabetic controls at 4 weeks is consistent with the endothelium-dependent impairment of vascular contractility reported in experimental T2DM [7,26]. This dysfunctional response is mechanistically attributed to hyperglycemia-induced generation of advanced glycation end products (AGEs), mitochondrial dysfunction, and activation of the polyol and hexosamine pathways, collectively impairing vascular smooth muscle function [27,28]. Notably, vitamin D3 treatment prevented the attenuation of maximal contractile responses at a submaximal phenylephrine concentration without altering sensitivity, as reflected in unaltered E_max_ and EC_50_ from concentration-response curves. This preservation of contractile amplitude, in the absence of a shift in potency, aligns with evidence that vitamin D3 deficiency exacerbates phenylephrine-induced contraction and that supplementation can restore contractile magnitude through improvements in endothelial NO availability [19,29,30]. The absence of significant differences in E_max_ and EC_50_ derived from the full concentration-effect curves is consistent with Santos et al. [31], who reported no changes in maximum phenylephrine response following vitamin D3 supplementation. Methodological differences across studies—including baseline tension, gas composition, and pharmacological inhibitors—may partly account for discrepancies with other reports [19].

Impaired endothelium-dependent relaxation is a hallmark of experimental T2DM and has been consistently reported in both macro- and microvascular preparations [19,32,33], although preserved responses have also been described under specific conditions [34]. Endothelium-dependent vasodilation involves coordinated release of relaxing factors—including NO, prostacyclin, and endothelium-derived hyperpolarizing factor (EDHF)—and its impairment may result from reduced EDRF production, increased NO inactivation, or enhanced generation of endothelium-derived constricting factors [35,36,37]. In the present study, vitamin D3 restored acetylcholine-induced relaxation to near-control levels at 4 weeks at both doses, consistent with [19,38]. The absence of this improvement at 8 weeks, particularly at the higher dose, likely reflects the progressive nature of diabetic endothelial damage under sustained hyperglycemia, and is in agreement with Santos et al. [31] and Mokhtar et al. [32]. Whether the enhanced relaxation at 4 weeks resulted from greater EDRF production or increased smooth muscle responsiveness remains to be determined, as endothelial mediators were not individually assessed.

The vasoprotective effects of vitamin D3 are primarily mediated through VDR activation in endothelial cells, leading to upregulation of eNOS, enhanced NO bioavailability, and attenuation of AGE-related endothelial injury and oxidative stress [29,39,40,41]. Furthermore, 1,25(OH)2D suppresses renin gene expression, thereby reducing RAAS activity and angiotensin II-mediated vasoconstriction [42,43,44,45,46,47,48,49,50]. At the immune-vascular interface, vitamin D3 promotes an anti-inflammatory milieu by upregulating IL-10 and downregulating TNF-α, IL-6, and COX-2, counteracting atherosclerotic progression [47,51,52]. In addition, 1,25(OH)2D modulates the expression of antithrombotic genes, reducing tissue factor and PAI-1 while increasing thrombomodulin and antithrombin [44,45,47,53]. These pleiotropic mechanisms collectively underpin the vasoprotective actions of vitamin D3 observed in the present study.

## 4. Materials and Methods

### 4.1. Animals and Ethical Aspects

To start the induction of experimental diabetes, male rats (Rattus norvergicus, Wistar strain), aged between 8 and 12 weeks and weighing between 180 and 200 g, from the Central Animal Facility of the Federal University of Piauí (UFPI), were used. The animals were kept in polypropylene boxes (3 to 4 rats/box), with standard feed (Nuvilab^®^ feed) and water ad libitum, in an air-conditioned room with an ambient temperature of 25 ± 2 °C and a photoperiod of 12 h light and 12 h dark. After the animals had acclimatized, they were randomly assigned to the groups described in the section on inducing diabetes mellitus and the experimental protocol.

The experimental protocol was approved by the Animal Use Ethics Committee (CEUA) of the Federal University of Piauí (opinion number 660/2020). The procedures followed the ethical principles set out in Law 11.794/08 [54] and Decree 6.899/09 [55], as well as Resolution 13/2013—National Council for Animal Care and Experimentation [56] and the Brazilian Guidelines for the Care and Use of Animals for Scientific and Didactic Purposes (DBCA/16) [57].

### 4.2. Induction of Diabetes Mellitus In Vivo

Induction of experimental diabetes was carried out using a protocol that combined ad libitum feeding of a high-fat, normoprotein diet (HFNPD) (Table 7) and intraperitoneal administration of streptozotocin (STZ) 30 mg/Kg, dissolved in 10 mM citrate buffer and pH 4.5, according to the methodology described by Oliveira et al. [58], with modifications. Table 7 shows the composition of the HFNPD used in this study. It should be noted that, unlike the study by Oliveira et al. [58], albumin was used instead of casein to correct the protein content. The chemical analysis of the standard feed and the HFNPD diet is described in Table 8.

During the first 24 h after induction, the animals had free access to a 10% glucose solution to avoid hypoglycemia. DM was confirmed 72 h after STZ injection by measuring fasting capillary blood glucose (12 h fast) using a portable glucometer and test strips (Accu-Chek Guide, Roche Diabetes Care, Basel, Switzerland). Fasting blood glucose values equal to or greater than 250 mg/dL were used as a criterion for confirming the presence of diabetes [59]. On the day the DM was confirmed, treatment was initiated.

### 4.3. In Vivo Experimental Groups

A total of 80 male Wistar rats were used as the final sample size for each group (n = 7–9), resulting from the exclusion of animals that did not reach the glycemic threshold for DM2 induction or mortality related to the severity of the diabetic model, ensuring only successfully induced animals were analyzed. The animals used in the study were initially randomly divided into two groups, the first consisting of 16 animals for the normal control group and the other with 64 animals for diabetes induction. The animals were divided into the experimental groups shown in Table 9:

### 4.4. Endothelial Function and Vascular Reactivity Ex Vivo in Response to Vasoconstrictor and Vasorelaxant Agents

#### 4.4.1. Solutions and Chemical Substances

Aortic function was measured ex vivo in Krebs solution containing (in mM): NaCl (118.0); KCl (4.6); CaCl_2_·2H_2_O (2.5); MgSO_4_·7H_2_O (5.7); NaHCO_3_ (25.0); KH_2_PO_4_·H_2_O (1.1); and D-glucose (11.0). The solution was prepared immediately before use; the temperature was maintained at 37 °C. Stable pH was achieved by using a gas mixture (containing O_2_ 21%, CO_2_ 5% and N_2_ 74%) [60]. Phenylephrine (Phe) was used as the contractile agent, and the muscarinic agonist acetylcholine (Ach) was used to investigate relaxation mediated by stimulation of the endothelium. Both the agonists Ach and Fen were purchased from Sigma-Aldrich (St Louis, MO, USA) and diluted in distilled water at the stock concentration [0.1 mol/L]. Volume additions corresponded to a maximum of 10% of the tank volume.

#### 4.4.2. Preparation of Aortic Artery Rings for Vascular Reactivity Testing

At the end of the treatment period (4 or 8 weeks), the animals were euthanized with thiopental (150 mg/Kg, intraperitoneal) to isolate and remove the descending segment of the thoracic aorta. After dissection and removal, the aortic segment was sectioned into rings, each approximately 5.0 mm long, according to the methodology described by Arcanjo et al. [60]. The rings were then suspended in vats in an isolated organ bath (MOD IOB2014003 from AVS Projetos, São Carlos, Brazil) using stainless steel hooks and cotton thread, one end of which was attached to force transducers (AQCAD 2.3.8., AVS Projetos, São Carlos, Brazil) coupled to a data acquisition system to record isometric tension, under a stabilization tension of 1 gf. After 45 min of stabilization, the vascular response to the substances tested was evaluated. The presence of endothelium was determined by applying 10 µM ACh to the bath containing the aortic segments previously contracted with 1 µM Fen. The number of ‘4’ rat aortic rings per animal were obtained and subjected to evaluation of endothelial integrity by a relaxation response ≥50% to acetylcholine (ACh, 10^−5^ M) in rings pre-contracted with phenylephrine (PHE, 10^−5^ M). Preparations failing to meet this criterion were excluded.

In order to evaluate the responses observed in the functional tests, the contractile effect mediated by the administration of Phe in each of the concentrations was calculated by the difference (i.e., Δ_Tension_ in grams) between the basal tension and the final tension obtained after the addition of Fen. The relaxation results mediated by ACh concentrations were expressed as relaxation percentage (%) from the maximum contraction response obtained by Phe (3 × 10^−7^ M) in order to normalize the data obtained (Table 10). From these experiments, the pharmacological parameters E_max_ (maximum effect) and the half-maximum effective concentration (EC_50_) were determined, which represent the concentration of agonists needed to induce a biological response corresponding to 50% of the maximum effect.

### 4.5. Statistical Analysis

Statistical analysis was carried out using the paired t-test or Analysis of Variance (ANOVA) followed by Tukey’s post-test for multiple comparisons between the groups. Prior to these tests, the normality of the data distribution was verified using the Shapiro–Wilk test, and the equality of variances was assessed using the Brown–Forsythe test. All data met the assumptions for parametric analysis (*p* > 0.05). As for the E_máx_/EC_50_ values and plotting of the vascular reactivity curves, these were calculated from the non-linear regression of the data used to assemble the concentration-response curves. Values were considered significant when *p* < 0.05. The tests were carried out using GraphPad Prism software version 8.0 (GraphPad Software, Inc., San Diego, CA, USA).

## 5. Conclusions

This study showed that vitamin D3 improved endothelial function, as acetylcholine-induced endothelium-dependent relaxation was significantly increased in the aortas of diabetic rats treated with vitamin D3 at both doses tested compared to diabetic control rats without any treatment in the 4-week experimental trial period. However, further studies aimed at elucidating the molecular mechanisms underlying the interaction between vitamin D3 and endothelial function are needed and are of crucial importance for the future management of diabetes complications.

## Figures and Tables

**Table 1:** Evaluation of glycemic parameters in Wistar rats with type 2 diabetes mellitus treated with vitamin D3 (0.25 and 0.50 µg/kg/day) for 4 weeks.

Groups	CN1 (n = 7)	CD1 (n = 7)	HIPO1 (n = 7)	VIT D1 0.25 (n = 7)	VIT D1 0.50 (n = 7)
Initial weight (g)	309.40 ± 6.30	290.60 ± 9.32	311.70 ± 3.77	285.00 ± 11.24	289.60 ± 7.28
Final weight (g)	351.60 ± 5.92 ^d,e^	325.00 ± 15.15	337.00 ± 5.99	296.60 ± 17.58 ^a^	295.90 ± 10.70 ^a^
Weight gain (g)	42.14 ± 3.97 ^e^	34.43 ± 7.33 ^e^	25.29 ± 4.88	20.83 ± 8.49	6.28 ± 6.07 ^a,b^
Glycated hemoglobin (%)	5.87 ± 0.52	7.95 ± 1.49	7.37 ± 1.65	5.70 ± 0.44	6.10 ± 0.43
Fasting blood glucose (mg/dL)	99.29 ± 2.35 ^b,c,d,e^	513.90 ± 17.98 ^a,d,e^	459.00 ± 9.55 ^a^	410.60 ± 28.55 ^a,b^	403.90 ± 31.68 ^a,b^

Mean ± SEM (Standard Error of the Mean). One Way ANOVA followed by Tukey’s test. Significant differences are presented as: ^a^ *p* < 0.05 when compared to CN1; ^b^ *p* < 0.05 when compared to CD1; ^c^ *p* < 0.05 when compared to HIPO1; ^d^ *p* < 0.05 when compared to VIT D1 0.25; ^e^ *p* < 0.05 when compared to VIT D2 0.50. CN1 = normal control treated for 4 weeks; CD1 = diabetic control treated for 4 weeks; HIPO1 = metformin treated for 4 weeks; VIT D1 0.25 = vitamin D3 dose 0.25 µg treated for 4 weeks; VIT D1 0.50 = vitamin D3 dose 0.50 µg treated for 4 weeks.

**Table 2:** Evaluation of glycemic parameters in Wistar rats with type 2 diabetes mellitus treated with vitamin D3 (0.25 and 0.50 µg/kg/day) for 8 weeks.

Groups	CN2 (n = 9)	CD2 (n = 9)	HIPO2 (n = 9)	VIT D2 0.25 (n = 9)	VIT D2 0.50 (n = 9)
Initial weight (g)	340.6 ± 11.13	338.1 ± 7.81	334.3 ± 14.18	343.6 ± 11.17	359.8 ± 13.10
Final weight (g)	375.0 ± 9.38	366.8 ± 8.73	376.6 ± 11.51	391.4 ± 10.38	402.3 ± 9.9
Weight gain (g)	34.38 ± 4.56	27.50 ± 4.67	42.38 ± 7.23	47.86 ± 5.54	43.00 ± 5.48
Glycated hemoglobin (%)	5.99 ± 1.21 ^b,c^	8.22 ± 0.66 ^a,e^	7.90 ± 0.37 ^a,e^	7.58 ± 1.95 ^e^	5.17 ± 1.28 ^b,c,d^
Fasting blood glucose (mg/dL)	82.00 ± 2.56 ^b,c,d,e^	454.20 ± 19.91 ^a,d^	419.10 ± 15.24 ^a^	383.30 ± 13.14 ^a,b^	401.70 ± 16.47 ^a^

Mean ± SEM (Standard Error of the Mean). One Way ANOVA followed by Tukey’s test. Significant differences are presented as: ^a^ *p* < 0.05 when compared to CN2; ^b^ *p* < 0.05 when compared to CD2; ^c^ *p* < 0.05 when compared to HIPO2; ^d^ *p* < 0.05 when compared to VIT D2 0.25; ^e^ *p* < 0.05 when compared to VIT D2 0.50. CN2 = normal control treated for 4 weeks; CD2 = diabetic control treated for 4 weeks; HIPO2 = metformin treated for 4 weeks; VIT D2 0.25 = vitamin D3 dose 0.25 µg treated for 4 weeks; VIT D2 0.50 = vitamin D3 dose 0.50 µg treated for 4 weeks.

**Table 3:** E_max_ and EC_50_ values determined from the concentration-effect curves for the cumulative addition of phenylephrine concentrations in isolated thoracic aortic rings of rats treated with vitamin D3 (0.25 and 0.50 µg/kg/day) for 4 weeks, analyzed by the variation in tension (%) and in grams of contraction.

Groups	Tension Variation (%)		Contraction (g)	
	E_max_ (%)	EC_50_ (M)	E_max_ (g)	EC_50_ (M)
CN1 (n = 20)	122.40 ± 8.93	5.79 × 10^−7^ ± 3.71 × 10^−7^	1.07 ± 0.21	5.51 × 10^−7^ ± 3.95 × 10^−7^
CD1 (n = 18)	126.80 ± 12.73	3.12 × 10^−6^ ± 2.78 × 10^−6^	0.95 ± 0.07 ^c^	3.77 × 10^−6^ ± 3.39 × 10^−6^
HIPO1 (n = 20)	132.60 ± 7.98	8.46 × 10^−7^ ± 2.82 × 10^−7^	0.74 ± 0.05 ^b^	8.47 × 10^−7^ ± 2.82 × 10^−7^
VIT D1 0.25 (n = 18)	109.30 ± 4.71	1.93 × 10^−7^ ± 9.56 × 10^−8^	0.98 ± 0.10	1.93 × 10^−7^ ± 9.56 × 10^−8^
VIT D1 0.50 (n = 18)	107.50 ± 2.73	1.98 × 10^−7^ ± 4.27 × 10^−8^	0.73 ± 0.06	1.97 × 10^−7^ ± 4.28 × 10^−8^

Mean ± SEM (Standard Error of the Mean). Unpaired *t*-test/One Way ANOVA followed by Tukey’s test. Significant differences are presented as: ^b^ *p* < 0.05 when compared to CD1; ^c^ *p* < 0.05 when compared to HIPO1. CN1 = non-diabetic rats treated for 4 weeks; CD1 = diabetic rats not treated for 4 weeks; HIPO1 = diabetic rats treated with metformin for 4 weeks; VIT D1 0.25 = diabetic rats treated with vitamin D3 at a dose of 0.25 µg/kg for 4 weeks; VIT D1 0.50 = diabetic rats treated with vitamin D3 at a dose of 0.50 µg/kg for 4 weeks. Legend: E_max_ (maximum drug effect) and EC_50_ (effective concentration at 50% of E_max_).

**Table 4:** E_max_ and EC_50_ values determined from the concentration-effect curves for the cumulative addition of phenylephrine concentrations in isolated thoracic aortic rings of rats treated with vitamin D3 (0.25 and 0.50 µg/kg/day) for 8 weeks, analyzed by the variation in tension (%) and in grams of contraction.

Groups	Tension Variation (%)		Contraction in Grams	
	E_max_ (%)	EC_50_ (M)	E_max_ (g)	EC_50_ (M)
CN2 (n = 22)	139.00 ± 10.12	1.52 × 10^−6^ ± 7.95 × 10^−7^	0.74 ± 0.06	1.52 × 10^−6^ ± 7.97 × 10^−7^
CD2 (n = 22)	135.30 ± 14.71	1.62 × 10^−6^ ± 9.79 × 10^−7^	0.66 ± 0.06	1.62 × 10^−6^ ± 9.83 × 10^−7^
HIPO2 (n = 22)	135.10 ± 9.60	1.09 × 10^−6^± 3.24 × 10^−7^	0.58 ± 0.03	1.09 × 10^−6^± 3.24 × 10^−7^
VIT D2 0.25 (n = 21)	127.50 ± 11.74	1.23 × 10^−6^ ± 9.37 × 10^−7^	0.64 ± 0.07	1.16 × 10^−6^ ± 8.17 × 10^−7^
VIT D2 0.50 (n = 20)	152.30 ± 16.40	2.43 × 10^−6^ ± 1.26 × 10^−6^	0.70 ± 0.10	2.43 × 10^−6^ ± 1.26 × 10^−6^

Mean ± SEM (Standard Error of the Mean). Unpaired *t*-test/One Way ANOVA followed by Tukey’s test. CN2 = non-diabetic rats treated for 8 weeks; CD2 = diabetic rats not treated for 8 weeks; HIPO2 = diabetic rats treated with metformin for 8 weeks; VIT D2 0.25 = diabetic rats treated with vitamin D3 at a dose of 0.25 µg/kg for 8 weeks; VIT D2 0.50 = diabetic rats treated with vitamin D3 at a dose of 0.50 µg/kg for 8 weeks. Legend: E_max_ (maximum drug effect) and EC_50_ (effective concentration at 50% of E_max_).

**Table 5:** E_max_ and EC_50_ values determined from the concentration-effect curves for the cumulative addition of acetylcholine concentrations in isolated thoracic aortic rings of rats treated with vitamin D3 (0.25 and 0.50 µg/kg/day) for 4 weeks, analyzed by tension variation (%) and relaxation in grams.

Groups	Tension Variation (%)		Relaxation in Grams	
	E_max_ (%)	EC_50_ (M)	E_max_ (g)	EC_50_ (M)
CN1 (n = 22)	117.14 ± 9.28 ^c^	4.18 × 10^−6^ ± 8.98 × 10^−7^	0.59 ± 0.04 ^c^	4.18 × 10^−6^ ± 8.98 × 10^−7^
CD1 (n = 20)	82.25 ± 13.84 ^a^	4.27 × 10^−6^ ± 2.22 × 10^−6^	0.30 ± 0.03 ^a^	4.27 × 10^−6^ ± 2.22 × 10^−6^
HIPO1 (n = 18)	76.26 ± 8.97	4.28 × 10^−6^ ± 1.55 × 10^−6^	0.33 ± 0.02	5.03 × 10^−6^ ± 1.33 × 10^−6^
VIT D1 0.25 (n = 20)	94.41 ± 6.59	2.96 × 10^−6^ ± 7.96 × 10^−7^	0.49 ± 0.05 ^b,c^	3.52 × 10^−6^ ± 9.44 × 10^−7^
VIT D1 0.50 (n = 22)	137.80 ± 13.87 ^b,c,d^	1.23 × 10^−5^ ± 5.34 × 10^−6^	0.41 ± 0.04 ^a^	1.23 × 10^−5^ ± 5.34 × 10^−6^

Mean ± SEM (Standard Error of the Mean). Unpaired t-test/One Way ANOVA followed by Tukey’s test. Significant differences are presented as: ^a^ *p* < 0.05 when compared to CN1; ^b^ *p* < 0.05 when compared to CD1; ^c^ *p* < 0.05 when compared to HIPO1; ^d^ *p* < 0.05 when compared to VIT D1 0.25; CN1 = non-diabetic rats treated for 4 weeks; CD1 = diabetic rats not treated for 4 weeks; HIPO1 = diabetic rats treated with metformin for 4 weeks; VIT D1 0.25 = diabetic rats treated with vitamin D3 at a dose of 0.25 µg/kg for 4 weeks; VIT D1 0.50 = diabetic rats treated with vitamin D3 at a dose of 0.50 µg/kg for 4 weeks. Legend: E_max_ (maximum drug effect) and EC_50_ (effective concentration at 50% of E_max_).

**Table 6:** E_max_ and EC_50_ values determined from the concentration-effect curves for the cumulative addition of acetylcholine concentrations in isolated thoracic aortic rings of rats treated with vitamin D3 (0.25 and 0.50 µg/kg/day) for 8 weeks, analyzed by voltage variation (%) and relaxation in grams.

Groups	Tension Variation (%)		Relaxation in Grams	
	E_max_ (%)	EC_50_ (M)	E_max_ (%)	EC_50_ (M)
CN2 (n = 22)	110.60 ± 8.85	5.50 × 10^−6^ ± 2.10 × 10^−6^	0.56 ± 0.04	5.54 × 10^−6^ ± 2.19 × 10^−6^
CD2 (n = 19)	73.52 ± 4.80 ^a,c^	2.29 × 10^−6^ ± 6.22 × 10^−7^	0.25 ± 0.01 ^a,c,d^	2.28 × 10^−6^ ± 6.72 × 10^−7^
HIPO2 (n = 21)	94.56 ± 6.90	3.64 × 10^−6^ ± 1.04 × 10^−6^	0.44 ± 0.06	4.66 × 10^−6^ ± 9.63 × 10^−7^
VIT D2 0.25 (n = 20)	88.47 ± 7.25	2.66 × 10^−6^ ± 6.94 × 10^−7^	0.46 ± 0.04	3.25 × 10^−6^ ± 8.41 × 10^−7^
VIT D2 0.50 (n = 20)	80.63 ± 6.85 ^a^	2.11 × 10^−6^ ± 1.04 × 10^−6^	0.39 ± 0.03 ^a^	1.79 × 10^−6^ ± 3.89 × 10^−7^

Mean ± SEM (Standard Error of the Mean). Unpaired *t*-test/One Way ANOVA followed by Tukey’s test. Significant differences are presented as: ^a^ *p* < 0.05 when compared to CN2; ^c^ *p* < 0.05 when compared to HIPO2; ^d^ *p* < 0.05 when compared to VIT D2 0.25; CN2 = non-diabetic rats treated for 8 weeks; CD2 = diabetic rats not treated for 8 weeks; HIPO2 = diabetic rats treated with metformin for 8 weeks; VIT D2 0.25 = diabetic rats treated with vitamin D3 at a dose of 0.25 µg/kg for 8 weeks; VIT D2 0.50 = diabetic rats treated with vitamin D3 at a dose of 0.50 µg/kg for 8 weeks. Legend: E_max_ (maximum drug effect) and EC_50_ (effective concentration at 50% of E_max_).

**Table 7:** Composition of the high-fat and normoprotein diets in g/100 g of feed.

Ingredients	Portion (g/100 g)
Nuvilab^®^ commercial feed	58.6
Lard	14.6
Crystal sugar	20.0
Albumin	6.6

**Table 8:** Centesimal composition of the standard diet (SD) and the high-fat, normoprotein diet (HFNPD), on a dry basis.

Component	SD	HFNPD
Humidity (%)	8.43 ± 0.11	4.01 ± 0.03 *
Ashes (%)	3.31 ± 0.09	4.48 ± 0.01 *
Lipids (%)	4.01 ± 0.02	17.91 ± 0.28 *
Proteins (%)	20.84 ± 0.34	19.70 ± 0.22
Carbohydrates ^#^ (%)	63.41± 0.44	53.90 ± 0.45 *

Mean ± standard error of the mean. ^#^ Carbohydrates calculated by difference, including fiber. * *p* < 0.05 in relation to SR. Unpaired *t*-test.

**Table 9:** Experimental groups and in vivo treatments to which the animals were subjected during the experimental trial period of 4 or 8 weeks.

Groups	Treatments
Normal control (NC) (n = 16), divided into: CN1—experimental trial for 4 weeks (n = 7) CN2—experimental trial for 8 weeks (n = 9)	Groups of animals not subjected to diabetes induction received Nuvilab^®^ commercial feed and treated with vehicle only (sunflower oil) during 4 or 8 weeks of experimental trial.
Diabetic control (DC) divided into: CD1—experimental trial for 4 weeks (n = 7) CD2—experimental trial for 8 weeks (n = 9)	Group in which diabetes was induced with animals with free access to HFNPD for 35 days, with administration of STZ 30 mg/kg in 10 mM citrate buffer and pH 4.5 intraperitoneally on the 36th day, not submitted to any treatment, only with vehicle (sunflower oil), maintained for 4 or 8 weeks of experimental testing.
Hypoglycemic group (HIPO) divided into: HIPO1—experimental trial for 4 weeks (n = 7) HIPO2—experimental trial for 8 weeks (n = 9)	Group in which diabetes was induced with animals with free access to HFNPD for 35 days, with administration of STZ 30 mg/kg in 10 mM citrate buffer and pH 4.5 intraperitoneally on the 36th day and treated with metformin at a dose of 150 mg/Kg/day for 4 or 8 weeks of the experimental trial.
VIT D 0.25 divided into: VIT D1 0.25—experimental trial for 4 weeks (n = 7) VIT D2 0.25—experimental trial for 8 weeks (n = 9)	Group in which diabetes was induced with animals with free access to HFNPD for 35 days, with administration of STZ 30 mg/kg in 10 mM citrate buffer and pH 4.5 intraperitoneally on the 36th day and treated with vitamin D3 at a dose of 0.25 µg/kg/day orally for 4 or 8 weeks of the experimental trial.
VIT D 0.50 divided into: VIT D1 0.50—experimental trial for 4 weeks (n = 7) VIT D2 0.50—experimental trial for 8 weeks (n = 9)	Group in which diabetes was induced with animals with free access to HFNPD for 35 days, with administration of STZ 30 mg/kg in 10 mM citrate buffer and pH 4.5 intraperitoneally on the 36th day and treated with vitamin D3 at a dose of 0.50 µg/kg/day orally for 4 or 8 weeks of the experimental trial.

**Table 10:** Equations for evaluating the responses observed in the functional tests of aortic segments from Wistar rats with type 2 diabetes mellitus.

Responses	Equations	
Vasoconstriction	$\frac{\left(Tx-Tbasal\right)}{Tmax-Tbasal}\times 100$	Tgrams − Tbasal grams
Vasorelaxation	$\frac{ACH-PHE}{PHE}\;\times 100$	ACH − PHE

## Data Availability

The data presented in this study are available on request from the corresponding author. The data are not publicly available due to privacy reasons.
